# CDC Health Information for International Travel 2016

**DOI:** 10.4269/ajtmh.16-0627

**Published:** 2016-11-02

**Authors:** Christian T. K.-H. Stadtländer

**Affiliations:** 1 Independent Researcher, St. Paul, MN 55125. E-mail: ctkstadtlander@msn.com

***CDC Health Information for International Travel 2016.*** Centers for Disease Control and Prevention, Gary W. Brunette (Ed.-in-Chief), Phyllis E. Kozarsky (Chief Med. Ed.), Nicole J. Cohen, Mark D. Gershman, Alan J. Magill, Stephen M. Ostroff, Edward T. Ryan, David R. Shlim, Michelle Weinberg, and Mary Elizabeth Wilson (Med. Eds.), 2016. 688 pp. New York, NY: Oxford University Press. US$49.95, ISBN: 978-0-19-937915-6

We live today in a world which can be characterized by a rapidly rising population, an unprecedented level of global connectedness, changes in international migration patterns, and a significant increase in international, business, and leisure travel. The United Nations reports that the world population reached 7.3 billion in mid-2015 and projects an increase to 8.5 billion in 2030.^1^ Business and trade today are truly international, and tourism is a global industry. According to the newest report of the World Tourism Organization, “In 2015, international tourism marked an impressive six consecutive years of above-average growth in terms of international tourist arrivals, with a record total of 1,184 million tourists travelling the world.”^2^ Yet, as important as international travel has become, it can pose risks to the health of the traveler and those who come in contact with the traveler. The Centers for Disease Control and Prevention (CDC) has recently published its 2016 edition of *CDC Health Information for International Travel*, also known as “The Yellow Book,” a continued valuable resource for those caring for international travelers.^3^

The book is divided into eight chapters which describe various aspects of international travel health, which will be discussed below. The book also offers three valuable appendices with an outline of the value of travel medicine (Appendix A), a list of available “travel vaccines” (Appendix B), and resources for migrant health (Appendix C). The Yellow Book is also available for purchase from e-book providers.

In Chapter 1 (Introduction), Lee and Kozarsky emphasize that “Travelers are as unique as their itineraries, covering all age ranges and having a variety of preexisting health concerns and conditions. The infectious disease risks that travelers face are dynamic―some travel destinations have become safer, whereas in other areas, new diseases have emerged and other diseases have reemerged.” This chapter contains CDC contact information, information on CDC travelers' health websites (including their images), and mobile applications such as “TravWell” and “Can I Eat This?” There are also sections, written by other authors, which deal with travel epidemiology, the role of the traveler in translocation of disease, and an interesting discussion about how travel guidelines are created and why they differ.

The second chapter is about “Pre-Travel Consultation.” As Chen and others point out, this consultation is important because it can help travelers understand potential health hazards, become aware of anticipated risks, and learn about methods of protection. In short, it is about assessing both the objective risk and the traveler's perceived risk, and about learning how to manage risk. In this chapter, Kroger and Strikas provide general recommendations for vaccination, including minimum ages and timing of vaccination, and allergy concerns. A discussion about the interactions among travel vaccines and drugs is provided by Youngster and Barnett. Other topics include travelers' diarrhea, water disinfection techniques, food poisoning precautions, and protection against arthropod-borne diseases. The authors also discuss other topics including the impact of jet lag, altitude and motion sickness, sun exposure, and animal-associated hazards, to name a few. I found the discussion about pretravel consultations well written and important because proper risk assessment is essential as it can help travelers avoid serious problems during the trip.

Chapter 3 is the largest chapter, covering infectious diseases related to travel. It describes over 70 diseases listed in alphabetical order, beginning with amebiasis and ending with Zika. The authors of each of these sections discuss the infectious agent(s), transmission routes, and epidemiology, as well as the clinical presentation and diagnosis of each disease, followed by treatment and prevention options. The book covers diseases that are endemic in many travel areas (e.g., malaria in Africa, Latin America, and southeast Asia) and infections that occur worldwide (e.g., human immunodeficiency virus [HIV] infection). Furthermore, the authors discuss diseases that can cause seasonal and pandemic outbreaks (e.g., influenza), emerging diseases (e.g., Zika), and recently resurging diseases (e.g., yellow fever). I believe that these sections are especially useful to the reader because those infections are often talked about in the media, and detailed information about them can guide travel health providers and, in turn, help travelers understand the seriousness of these infectious diseases. A critique I have is the positioning of “Yellow Fever and Malaria Information, by Country” (pp. 365–420) after the discussion of Zika. Although I like the presentation of the information in form of text, tables, and high-quality colored maps, presenting this section as an Appendix would have been perhaps a better choice.

In the following chapter (Chapter 4), the authors discuss issues associated with specific travel destinations. Shlim mentions that this chapter was designed to help providers “feel more comfortable giving advice about specific destinations that he or she may have never visited, and to provide a level of detail about the attractions and health risks at select destinations that has not been provided elsewhere in this book.” Specific destinations presented by the authors include Africa, the Americas, the Caribbean, Asia, and the Middle East. “Post-Travel Evaluation” is the topic of the fifth chapter. Here, Fairley points out that “Most post-travel infections become apparent soon after travel, but because incubation periods vary, some syndromes can present months to years after initial infection.” The authors of this chapter address issues such as fever in returned travelers, persistent travelers' diarrhea, and skin and soft tissue infections. They also discuss the screening of asymptomatic returned travelers for occult parasitic infections and nonparasitic illnesses (e.g., HIV and hepatitis B virus infections). These two chapters are valuable because travelers typically select destinations based on their attractions and not necessarily based on avoiding health risk.

The following chapter (Chapter 6) is about conveyance and transportation issues. Illig and others mention that travel by commercial aircraft is expected to double in the next 20 years (currently more than 1 billion people choose air travel every year). Immobility during flights (risk of thromboembolic disease), close proximity to other passengers with certain communicable diseases, and exacerbations of chronic medical problems are discussed. Other sections deal with cruise ship travel and associated risks of outbreaks of, for example, noroviral gastrointestinal infections and respiratory infections (influenza and Legionnaires' disease). There are also discussions on what one needs to know in case death occurs during travel and when taking animals and animal products across international borders.

The seventh chapter is entitled “International Travel with Infants and Children.” Here, Weinberg and others emphasize that there has been a significant increase in the number of children who travel or live outside their home countries. The authors review the most commonly reported health problems among child travelers, including diarrheal illnesses, dermatologic conditions (e.g., those caused by animal and arthropod bites), systemic febrile illnesses (especially malaria), and respiratory disorders. Other authors provide vaccine recommendations for infants and children, discuss traveling with a breastfeeding child, and address issues associated with international child adoption (e.g., underimmunization and absence of a complete medical history).

In the final chapter (Chapter 8), the authors give advice for travelers who have specific needs. Topics include travelers with chronic illnesses, travelers who are pregnant, and those with disabilities. There are also sections on airline crews, international student travelers, military personnel, long-term travelers, and immigrants and refugees, among others. I like that the authors covered here a large number of travelers with specific needs because providing guidance for reducing risk in any type of traveler is what this Yellow Book is all about.

In my opinion, this is an excellent book. It is well written, sufficiently referenced, and superbly illustrated. I like that the editors included sections called “Perspectives” and “For the Record,” in which individual topics are further explored. An example of a perspective is “Fear of Vaccines,” and of a record is “A History of Polio Eradication.” It was mentioned in the Introduction that the Yellow Book was primarily written for clinicians, but others working in the travel industry, volunteer organizations, and multinational corporations, as well as missionaries and individual travelers may also find this book useful. I believe that we should add to this list researchers (especially microbiologists, epidemiologists, parasitologists, and virologists) and health administrators, as well as faculty and students in infectious disease, international health, travel medicine, and tropical medicine. I highly recommend this book.
